# *catena*-Poly[[bis­(pyrazine-2-carbox­amide-κ*N* ^4^)mercury(II)]-di-μ-bromido]

**DOI:** 10.1107/S1600536810001182

**Published:** 2010-01-16

**Authors:** Bahareh Mir Mohammad Sadegh, Alireza Azhdari Tehrani, Hamid Reza Khavasi

**Affiliations:** a Department of Chemistry, Shahid Beheshti University, G. C., Evin, Tehran 1983963113, Iran

## Abstract

In the crystal structure of the title compound, [HgBr_2_(C_5_H_5_N_3_O)_2_]_*n*_, the Hg^II^ cation is located on an inversion center and is coordinated by two N atoms from the pyrazine rings and four bridging Br^−^ anions in a distorted octa­hedral geometry. The Br^−^ anions bridge the Hg^II^ cations with significantly different Hg—Br bond distances of 2.4775 (8) and 3.1122 (8) Å, forming polymeric chains running along the *a* axis. Inter­molecular N—H⋯O and N—H⋯N hydrogen bonds are effective in the stabilization of the crystal structure.

## Related literature

For metal-binding properties of pyridine and pyrazine ligands, see: Sasan *et al.* (2008 ▶); Khavasi *et al.* (2009 ▶); Petro & Mukherjee (1999 ▶); Sigh & Mukherjee (2005 ▶). For the coordination modes of pyrazine­amide, see: Hausmann & Brooker (2004 ▶); Cati & Stoeckli-Evans (2004 ▶); Miyazaki *et al.* (2007 ▶).
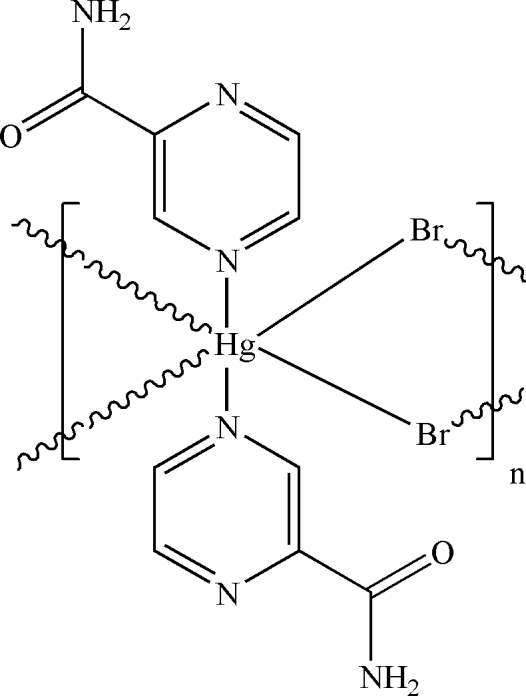


## Experimental

### 

#### Crystal data

[HgBr_2_(C_5_H_5_N_3_O)_2_]
*M* *_r_* = 606.63
Triclinic, 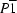

*a* = 3.9628 (5) Å
*b* = 6.5162 (9) Å
*c* = 15.0388 (19) Å
α = 101.783 (10)°
β = 93.418 (11)°
γ = 95.214 (11)°
*V* = 377.36 (9) Å^3^
*Z* = 1
Mo *K*α radiation
μ = 15.50 mm^−1^
*T* = 298 K
0.50 × 0.06 × 0.03 mm

#### Data collection

Stoe IPDS II diffractometer
Absorption correction: multi-scan (*X-RED* and *X-SHAPE*; Stoe & Cie, 2005 ▶) *T*_min_ = 0.345, *T*_max_ = 0.630
4311 measured reflections
2002 independent reflections
1933 reflections with *I* > 2σ(*I*)
*R*_int_ = 0.144

#### Refinement

*R*[*F* ^2^ > 2σ(*F* ^2^)] = 0.065
*wR*(*F* ^2^) = 0.173
*S* = 1.11
2002 reflections
97 parameters
H-atom parameters constrained
Δρ_max_ = 3.93 e Å^−3^
Δρ_min_ = −5.48 e Å^−3^

### 

Data collection: *X-AREA* (Stoe & Cie, 2005 ▶); cell refinement: *X-AREA*; data reduction: *X-AREA*; program(s) used to solve structure: *SHELXS97* (Sheldrick, 2008 ▶); program(s) used to refine structure: *SHELXL97* (Sheldrick, 2008 ▶); molecular graphics: *ORTEP-3 for Windows* (Farrugia, 1997 ▶); software used to prepare material for publication: *WinGX* (Farrugia, 1999 ▶).

## Supplementary Material

Crystal structure: contains datablocks global, I. DOI: 10.1107/S1600536810001182/xu2716sup1.cif

Structure factors: contains datablocks I. DOI: 10.1107/S1600536810001182/xu2716Isup2.hkl

Additional supplementary materials:  crystallographic information; 3D view; checkCIF report

## Figures and Tables

**Table 1:** Selected bond lengths (Å)

Hg1—Br1	2.4775 (8)
Hg1—Br1^i^	3.1122 (8)
Hg1—N2	2.758 (6)

Symmetry code: (i) 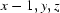.

**Table 2:** Hydrogen-bond geometry (Å, °)

*D*—H⋯*A*	*D*—H	H⋯*A*	*D*⋯*A*	*D*—H⋯*A*
N3—H3*A*⋯O1^ii^	0.86	2.02	2.881 (11)	174
N3—H3*B*⋯N1^iii^	0.86	2.53	3.214 (11)	137

Symmetry codes: (ii) 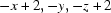; (iii) 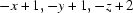.

**Fig. 1.**
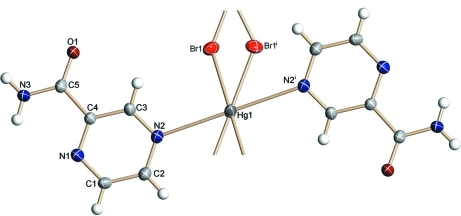
The molecular structure with the atom-numbering scheme. Displacement ellipsoids are drawn at 30% probability level.

**Fig. 2.**
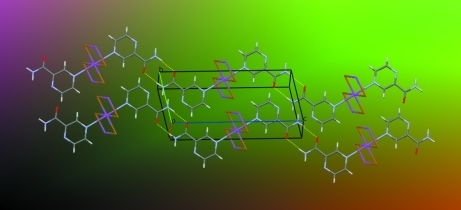
A packing diagram of (I). Hydrogen bonds are shown as dashed lines.

## supplementary crystallographic information

### Comment

A large variety of pyridine and pyrazine amide ligands have been synthesized for investigating their metal-binding properties (Sasan *et al.*, 2008; Khavasi *et al.*, 2009; Petro & Mukherjee, 1999; Sigh & Mukherjee, 2005). The coordination chemistry of parazineamides is rich. Examples of coordination *via* the pyrazine N atoms, the carbonyl O atoms and the amide N atoms of the ligand in a non-, mono-, or bis-deprotonated form are known (Hausmann & Brooker, 2004; Cati & Stoeckli-Evans, 2004; Miyazaki *et al.*, 2007) and metal complexes of the ligands have been used extensively to mimic the properties of biologically active systems. Here we synthesized the title compound, (I), and report here its crystal structure.

The asymmetric unit of the title compound, (I), contains one half-molecule (Fig. 1). The Hg^II^ atom is six-coordinated in a distorted octahedral configuration by two N atoms from pyrazine amides and four bridging Br atoms. The bridging function of bromo atoms leads to a one-dimensional chain structure. The Hg—Br and Hg—N bond lengths and angles (Table 1) are within normal ranges. In the crystal structure (Fig. 2), intermolecular N—H···O and N—H···N hydrogen bonds (Table 2) result in the formation of a supramolecular structure, in which they may be effective in the stabilization of the structure.

### Experimental

For the preparation of the title compound, a solution of pyrazineamide (0.246 g, 2.0 mmol) in methanol (10 ml) was added to a solution of HgBr_2_ (0.360 g, 1.0 mmol) in methanol (5 ml) at room temperature. The suitable crystals for X-ray analysis were obtained by slow evaporation from methanolic solution after one week (yield 0.500 g, 82.5%).

### Refinement

All of the H atoms were positioned geometrically with C–H = 0.93 and N—H = 0.86 Å and constrained to ride on their parent atoms, with *U*_iso_(H) = 1.2*U*_eq_(C,*N*). The largest peak and deepest hole are near to the Hg1 atom (0.90 and 0.79 Å, respectively).

### Crystal data

**Table:**

[HgBr_2_(C_5_H_5_N_3_O)_2_]	*Z* = 1
*M**_r_* = 606.63	*F*(000) = 278
Triclinic, *P*1	*D*_x_ = 2.669 Mg m^−^^3^
Hall symbol: -P 1	Mo *K*α radiation, λ = 0.71073 Å
*a* = 3.9628 (5) Å	Cell parameters from 765 reflections
*b* = 6.5162 (9) Å	θ = 3.2–29.1°
*c* = 15.0388 (19) Å	µ = 15.50 mm^−^^1^
α = 101.783 (10)°	*T* = 298 K
β = 93.418 (11)°	Needle, colorless
γ = 95.214 (11)°	0.5 × 0.06 × 0.03 mm
*V* = 377.36 (9) Å^3^	

### Data collection

**Table:**

Stoe IPDS II diffractometer	1933 reflections with *I* > 2σ(*I*)
rotation method scans	*R*_int_ = 0.144
Absorption correction: multi-scan (*X-RED* and *X-SHAPE*; Stoe & Cie, 2005)	θ_max_ = 29.1°, θ_min_ = 3.2°
*T*_min_ = 0.345, *T*_max_ = 0.630	*h* = −5→5
4311 measured reflections	*k* = −8→8
2002 independent reflections	*l* = −20→20

### Refinement

**Table:**

Refinement on *F*^2^	0 restraints
Least-squares matrix: full	H-atom parameters constrained
*R*[*F*^2^ > 2σ(*F*^2^)] = 0.065	*w* = 1/[σ^2^(*F*_o_^2^) + (0.1262*P*)^2^] where *P* = (*F*_o_^2^ + 2*F*_c_^2^)/3
*wR*(*F*^2^) = 0.173	(Δ/σ)_max_ < 0.001
*S* = 1.11	Δρ_max_ = 3.93 e Å^−^^3^
2002 reflections	Δρ_min_ = −5.48 e Å^−^^3^
97 parameters	

### Special details

**Table:**

Geometry. All e.s.d.'s (except the e.s.d. in the dihedral angle between two l.s. planes) are estimated using the full covariance matrix. The cell e.s.d.'s are taken into account individually in the estimation of e.s.d.'s in distances, angles and torsion angles; correlations between e.s.d.'s in cell parameters are only used when they are defined by crystal symmetry. An approximate (isotropic) treatment of cell e.s.d.'s is used for estimating e.s.d.'s involving l.s. planes.

### Fractional atomic coordinates and isotropic or equivalent isotropic displacement parameters (Å^2^)

**Table:**

	*x*	*y*	*z*	*U*_iso_*/*U*_eq_	
C1	0.402 (2)	0.5193 (13)	0.7914 (6)	0.0456 (17)	
H1	0.3215	0.6506	0.8064	0.055*	
C2	0.400 (2)	0.4210 (13)	0.7010 (6)	0.0444 (16)	
H2	0.3156	0.4876	0.6566	0.053*	
C3	0.629 (2)	0.1448 (13)	0.7407 (5)	0.0414 (15)	
H3	0.7049	0.0123	0.7252	0.05*	
C4	0.638 (2)	0.2438 (14)	0.8329 (6)	0.0381 (15)	
C5	0.790 (2)	0.1363 (13)	0.9029 (5)	0.0413 (15)	
N1	0.519 (2)	0.4278 (10)	0.8588 (5)	0.0420 (14)	
N2	0.5148 (18)	0.2350 (10)	0.6752 (4)	0.0422 (13)	
N3	0.779 (2)	0.2309 (12)	0.9885 (5)	0.0525 (17)	
H3A	0.8633	0.1766	1.0313	0.063*	
H3B	0.6883	0.3471	1.0017	0.063*	
O1	0.914 (2)	−0.0290 (12)	0.8783 (5)	0.0577 (19)	
Hg1	0.5	0	0.5	0.0390 (2)	
Br1	0.86218 (19)	−0.24778 (12)	0.55380 (6)	0.0394 (2)	

### Atomic displacement parameters (Å^2^)

**Table:**

	*U*^11^	*U*^22^	*U*^33^	*U*^12^	*U*^13^	*U*^23^
C1	0.065 (5)	0.036 (3)	0.038 (4)	0.015 (3)	−0.006 (3)	0.011 (3)
C2	0.055 (4)	0.042 (4)	0.037 (3)	0.004 (3)	−0.009 (3)	0.014 (3)
C3	0.056 (4)	0.039 (3)	0.030 (3)	0.013 (3)	−0.006 (3)	0.006 (3)
C4	0.048 (4)	0.037 (3)	0.028 (3)	0.006 (3)	−0.005 (3)	0.004 (3)
C5	0.055 (4)	0.041 (4)	0.029 (3)	0.011 (3)	−0.002 (3)	0.009 (3)
N1	0.062 (4)	0.031 (3)	0.032 (3)	0.007 (3)	−0.007 (3)	0.007 (2)
N2	0.057 (3)	0.040 (3)	0.029 (3)	0.007 (3)	−0.007 (2)	0.010 (2)
N3	0.085 (5)	0.045 (3)	0.030 (3)	0.023 (4)	−0.005 (3)	0.009 (3)
O1	0.095 (6)	0.049 (3)	0.031 (3)	0.032 (4)	−0.005 (3)	0.007 (2)
Hg1	0.0387 (3)	0.0433 (3)	0.0380 (3)	0.01479 (17)	−0.00149 (16)	0.01255 (19)
Br1	0.0390 (4)	0.0374 (4)	0.0453 (5)	0.0116 (3)	0.0000 (3)	0.0146 (3)

### Geometric parameters (Å, °)

**Table:**

C1—N1	1.356 (10)	C5—O1	1.224 (11)
C1—C2	1.378 (12)	C5—N3	1.313 (10)
C1—H1	0.93	N3—H3A	0.86
C2—N2	1.325 (11)	N3—H3B	0.86
C2—H2	0.93	Hg1—Br1	2.4775 (8)
C3—N2	1.323 (9)	Hg1—Br1^i^	2.4775 (8)
C3—C4	1.402 (11)	Hg1—Br1^ii^	3.1122 (8)
C3—H3	0.93	Hg1—Br1^iii^	3.1122 (8)
C4—N1	1.321 (12)	Hg1—N2	2.758 (6)
C4—C5	1.505 (12)	Hg1—N2^i^	2.758 (6)
			
N1—C1—C2	121.2 (8)	N3—C5—C4	116.1 (8)
N1—C1—H1	119.4	C4—N1—C1	116.5 (7)
C2—C1—H1	119.4	C3—N2—C2	116.8 (7)
N2—C2—C1	122.2 (7)	C5—N3—H3A	120
N2—C2—H2	118.9	C5—N3—H3B	120
C1—C2—H2	118.9	H3A—N3—H3B	120
N2—C3—C4	121.7 (8)	Br1—Hg1—Br1^i^	180.00 (4)
N2—C3—H3	119.1	Br1—Hg1—Br1^ii^	90.44 (2)
C4—C3—H3	119.1	Br1^i^—Hg1—Br1^ii^	89.56 (2)
N1—C4—C3	121.5 (8)	Br1—Hg1—Br1^iii^	89.56 (2)
N1—C4—C5	120.1 (7)	Br1^i^—Hg1—Br1^iii^	90.44 (2)
C3—C4—C5	118.4 (8)	Br1^ii^—Hg1—Br1^iii^	180.000 (17)
O1—C5—N3	124.2 (8)	Hg1—Br1—Hg1^iv^	89.56 (2)
O1—C5—C4	119.7 (7)		
			
N1—C1—C2—N2	−0.7 (15)	C3—C4—C5—N3	−177.2 (9)
N2—C3—C4—N1	2.9 (14)	C3—C4—N1—C1	−2.6 (12)
N2—C3—C4—C5	−176.4 (8)	C5—C4—N1—C1	176.7 (9)
N1—C4—C5—O1	−176.4 (8)	C2—C1—N1—C4	1.6 (13)
C3—C4—C5—O1	3.0 (14)	C4—C3—N2—C2	−1.9 (12)
N1—C4—C5—N3	3.4 (13)	C1—C2—N2—C3	0.8 (13)

Symmetry codes: (i) −*x*+1, −*y*, −*z*+1; (ii) −*x*+2, −*y*, −*z*+1; (iii) *x*−1, *y*, *z*; (iv) *x*+1, *y*, *z*.

### Hydrogen-bond geometry (Å, °)

**Table:**

*D*—H···*A*	*D*—H	H···*A*	*D*···*A*	*D*—H···*A*
N3—H3A···O1^v^	0.86	2.02	2.881 (11)	174
N3—H3B···N1^vi^	0.86	2.53	3.214 (11)	137

Symmetry codes: (v) −*x*+2, −*y*, −*z*+2; (vi) −*x*+1, −*y*+1, −*z*+2.
